# Safety assessment of coronary arteries during left bundle branch area pacing

**DOI:** 10.1007/s00059-024-05259-w

**Published:** 2024-08-05

**Authors:** Qiling Kong, Huolong Chen, Juan Hua, Ziyi Xiong, Shuyun Le, Jinwei Liu, Dandan Wang, Qi Chen

**Affiliations:** 1 Department of Cardiovascular Medicine, The Second Affiliated Hospital of Nanchang University, No. 1 Minde Rd, East-lake District, 330006 Nanchang, Jiangxi China; 2 Department of Cardiovascular Medicine, Wuhan Third Hospital, Tongren Hospital of Wuhan University, 430060 Wuhan, China

**Keywords:** Left bundle branch area pacing, Electrode, Coronary artery, Safety assessment, Pacemaker complication, Linksschenkelstimulation, Elektrode, Koronararterie, Sicherheitsbeurteilung, Schrittmacherkomplikation

## Abstract

**Background:**

This study aimed to assess the safety of left bundle branch area pacing (LBBAP) by measuring the distance from the tip of the electrode to the nearby coronary artery with a nine-partition grid method.

**Methods:**

From January 2019 to October 2020, patients who underwent LBBAP and postoperative coronary angiography in the Second Affiliated Hospital of Nanchang University were included in the study. The patients’ fluoroscopic images of LBBAP and coronary angiography were collected and analyzed. Changes in the ST‑T segment in the electrocardiogram (ECG), serum troponin, and myocardial enzyme profiles were observed before and after the LBBAP procedure.

**Results:**

A total of 50 patients were included in this study, of whom 46 patients underwent implantation with a pacemaker and 4 patients received an implantable cardioverter defibrillator (ICD). The pacing electrodes were confined to the posterior–middle (PM), median (M), Posterior inferior (PI), and middle inferior (MI) positions of the two-dimensional nine-square grid or in the junction area of the above positions, and were concentrated in the rectangle formed by the line of the center points of the four positions. The average vertical distances from the electrode tip to the left anterior descending branch artery (LAD), posterior descending branches (PD) and the left posterior ventricular branches (PL) were 19.69 ± 8.72 mm, 26.09 ± 8.02 mm, and 21.11 ± 7.86 mm, respectively; the minimum was 5.28 mm, 9.51 mm, and 8.69 mm, respectively. Coronary angiography in all patients showed no significant injury to the ventricular septal branch; however, we observed elevated serum troponin and changes in ST‑T segment in ECG.

**Conclusion:**

The study demonstrates that pacing electrodes in LBBAP can be safely implanted over a wide range. Coronary arteries are likely to be safe when the pacing electrodes are located within the rectangle formed by the line connecting the PM, M, PI, and MI zone centroids. The left bundle branch can be quickly captured and the safety of the coronary artery can be improved by locating the electrode in the posterior–mid zone. The potential risk of injury to the LAD from the electrode is greater compared with the PD.

## Abstract (de)

**Hintergrund:**

Ziel der vorliegenden Studie war es, die Sicherheit der Linksschenkelstimulation („left bundle branch area pacing“ [LBBAP]) durch Messung der Distanz von der Elektrodenspitze bis zur nahegelegenen Koronararterie unter Verwendung eines 9‑teiligen Gitters zu untersuchen.

**Methoden:**

In die Studie wurden Patienten einbezogen, bei denen zwischen Januar 2019 und Oktober 2020 eine LBBAP und eine postoperative Koronarangiographie im Second Affiliated Hospital of Nanchang University, Nanchang, China, durchgeführt wurde. Die Durchleuchtungsaufnahmen der Patienten von der LBBAP und der Koronarangiographie wurden erfasst und ausgewertet. Veränderungen im ST-T-Segment im Elektrokardiogramm (EKG), Serumtroponin und Myokardenzymprofil vor und nach der LBBAP wurden festgestellt.

**Ergebnisse:**

In die Studie wurden 50 Patienten einbezogen, bei 46 davon erfolgte eine Schrittmacherimplantation, und 4 Patienten erhielten einen implantierbaren Kardioverter-Defibrillator (ICD). Die Schrittmacherelektroden waren begrenzt auf die Positionen posterior–mittig (PM), median (M), posterior–inferior (PI), und mittig-inferior (MI) des zweidimensionalen 9‑Felder-Gitters oder im Kreuzungsbereich der genannten Positionen, sie waren konzentriert in einem Rechteck, das durch die Linie zwischen den zentralen Punkten der 4 Positionen gebildet wurde. Die durchschnittlichen vertikalen Distanzen von der Elektrodenspitze bis zu dem Ramus interventricularis anterior (RIVA), den Rami interventriculares posteriores (RIVP) und den Rami posteriores ventriculi sinistri betrugen 19,69 ± 8,72 mm, 26,09 ± 8,02 mm bzw. 21,11 ± 7,86 mm; das Minimum lag bei 5,28 mm, 9,51 mm bzw. 8,69 mm. Die Koronarangiographie ergab bei allen Patienten keine wesentliche Verletzung der Rami interventriculares septales; es wurden jedoch ein erhöhtes Serumtroponin und Veränderungen im ST-T-Segment des EKG festgestellt.

**Schlussfolgerung:**

Die vorliegende Studie zeigt, dass die Schrittmacherelektroden bei LBBAP in einem weiten Bereich sicher implantiert werden kann. Die Koronararterien sind wahrscheinlich sicher, wenn die Schrittmacherelektroden sich innerhalb des Rechtecks befinden, dass durch die Linie erzeugt wird, welche die zentralen Punkte der Zonen PM, M, PI und MI verbindet. Der Linksschenkel kann schnell erfasst werden, und die Sicherheit der Koronararterien kann durch Platzierung der Elektrode in der in der PM-Zone verbessert werden. Das potenzielle Risiko der Verletzung durch die Elektrode ist für RIVA höher als für RIVP.

## Methods

### Inclusion and exclusion criteria

This study collected the imaging information and data of inpatients who underwent the LBBAP procedure and coronary angiography (CAG) in the Center of Cardiovascular Medicine, the Second Affiliated Hospital of Nanchang University, from 1 January 2019 to 1 October 2020. This study was approved by the local ethical board and was performed in accordance with the Declaration of Helsinki.

### Inclusion criteria

The study included patients who underwent LBBAP surgery for one or more of the following conditions and underwent coronary angiography postoperatively, including (a) sick sinus syndrome, (b) atrioventricular block, (c) dilated cardiomyopathy, (d) hypertrophic cardiomyopathy and (e) ventricular tachycardia.

### Exclusion criteria

Inpatients with one or more of the following diseases were excluded: (a) acute myocardial infarction, (b) prior cardiac stent implantation, (c) chronic occlusive disease of the coronary arteries, and (d) coronary calcifications or anomalies.

### LBBAP procedure

In this study, all the pacemaker implantations were performed through the left subclavian vein or the left axillary vein. During surgery, a C315 His sheath was inserted into the tricuspid ring using a J-wire. In the right anterior oblique position (RAO), the pacemaker electrode (Model 3830, 69 cm, Medtronic Inc., Minneapolis, MN, USA) was pushed forward through the sheath, with the distal screw located at the tip of the catheter. First, the His potential was marked, and the tip of the pacing lead was moved downward and forward in the ventricular direction for approximately 1.5–2 cm.

Subsequently, successful LBBAP implantation met the following criteria: (a) The pacing QRS interval in the V1 lead was less than 130 ms or exhibited a W-shaped QRS complex. (b) The tip of the electrode was carefully screwed into the left ventricular septum while in the left anterior oblique (LAO) 30–45° position. (c) The V1 QRS complex, initially showing a W-shaped “notch,” gradually shifted until the R wave appeared late in the QRS complex. (d) The pacing pattern gradually transitioned from an LBBB pattern to an RBBB pattern.

Following successful implementation, the electrode was securely fixed and connected to the pacemaker using screws. During the implantation process, various assessments were conducted. These included monitoring the pacing threshold, recording the left bundle branch potential, monitoring pacing impedance, and performing concurrent 12-lead ECG recordings.

### Pacing electrode positions

**Fig. 1:**
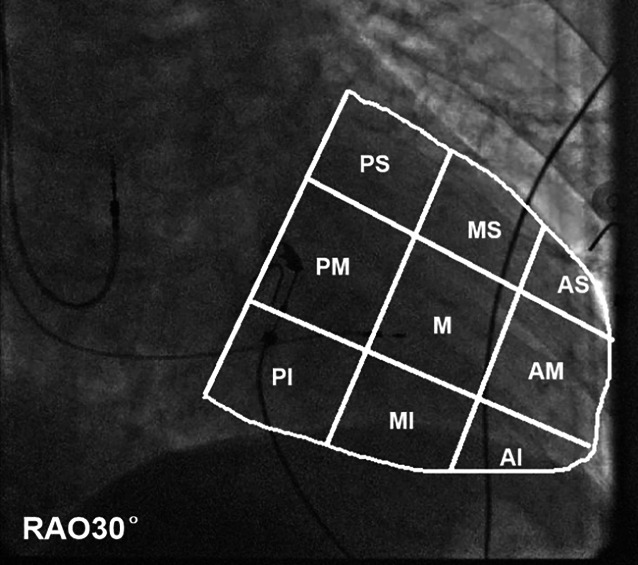
Distribution of nine-partition grid method in fluoroscopic image at right anterior oblique (RAO) 30°projection. *PS* posterior superior, *MS* middle superior, *AS* anterior superior, *PM* posterior median, *M* median, *AM* anterior median, *PI* posterior inferior, *MI* middle inferior, *AI* anterior inferior

Two nonsurgical physicians independently reviewed the patients’ imaging data and utilized the simplified nine-partition grid method [9] to record the distribution of pacing electrode positions. Figure 1 displays the fluoroscopic image of the entire ventricle silhouette, outlined by a white arc. In the RAO 30° view, the coronary sinus ostium (CSo) served as a crucial landmark. The atrioventricular boundary line passed through the CSo, nearly perpendicular to the leading edge line of the ventricle. The fluoroscopic image of the ventricle silhouette was divided into nine partitions, each partition being approximately equal in length.

From left to right and from top to bottom, the ventricles were marked as posterior–superior (PS), middle–superior (MS), anterior–superior (AS), posterior–median (PM), median (M), anterior–median (AM), posterior–inferior (PI), middle–inferior (MI), and anterior–inferior (AI). We observed and recorded the pacing electrode positions in the nine partitions using a two-dimensional projection position.

### Distance between the electrode and the coronary artery

**Fig. 2:**
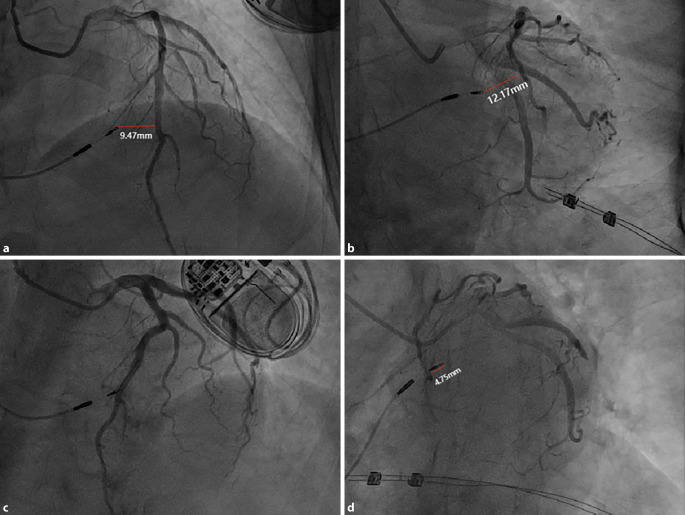
Vertical distance from the tip of the pacing electrode to the LAD during systole. **a** AP + CRA 30° 9.47 mm in fluoroscopic image. **b** AP + CAU 30° 12.17 mm in fluoroscopic image. **c** LAO 30° + CRA 20° in fluoroscopic image (the tip of the pacing electrode overlaps with the LAD; thus, the vertical distance is measured as 0 mm). **d** LAO 45° + CAU 30° 4.75 mm in fluoroscopic image; thus, the maximum distance is 12.17 mm

As shown in Fig. 2, we measured the vertical distance from the tip of the electrode to the LAD during systole, with the positive head position (AP + CRA 30°), foot position (AP + CAU 30°), left shoulder position (LAO 30° + CRA 20°), and spider position (LAO 45° + CAU 30°). The maximum value was recorded, which was closest to the actual spatial distance from the tip of the electrode to the LAD. The same method was used to measure the distance from the tip of the electrode to the PD and PL. The measurement results are represent using two decimal places.

### Other complications

With the cardiac silhouette excursion method [10], the occurrence of early tamponade was observed during the LBBAP procedure and echocardiography within 24 h of the procedure. Simultaneously, before and after pacemaker implantation, the ECG, serum troponin I (TnI), myoglobin (MB), and myocardial enzyme spectrum including creatine kinase (CK), creatine kinase isoenzymes (CK-MB), and lactate dehydrogenase (LDH) were tested to observe the myocardial injury and the blood vessels injured by pacing electrodes [11]. All patients had 5 mL of fasting venous blood drawn for testing. Cardiac enzyme spectrum indexes (CK, CK-MB, LDH) were detected with an automatic biochemical analyzer (Beckman Coulter AU5800, Brea, CA, USA); TnI and MB were determined with a fully automated chemiluminescence immunoassay analyzer (Beckman DIX800), and the detection operation was strictly in accordance with the relevant instructions of the kit to ensure the reliability of the test results.

### Statistical analysis

We used IBM SPSS Statistics software (version 20, Chicago, IL, USA) to analyze the data. For data with a normal distribution, the one-way repeated-measures analysis of variance (ANOVA) and Tukey’s post hoc test were used for multiple groups; otherwise, the Kruskal–Wallis’s test was used. Statistical significance was set at a two-tailed *p* < 0.05 value. The distance from the pacing electrode to the coronary artery was calculated as the mean ± standard deviation.

## Results

### Baseline characteristics

We selected 139 patients who underwent LBBAP in our hospital between January 2019 and October 2020. Among them, 89 patients with acute myocardial infarction and previous cardiac stent implantation were excluded, leaving 50 patients (27 men) for analysis. Regarding the indications of surgery, 38 were for atrioventricular block, 4 were for sick sinus syndrome, and 8 were for heart failure with non-ischemic cardiomyopathy. Four patients received VVIR for sustained atrial fibrillation with atrioventricular block. The QRS interval after the LBBAP procedure was significantly shorter than before (124.9 ± 32.6 ms vs. 113.4 ± 17.1 ms, *p* *<* 0.05). The baseline characteristics are shown in Table 1.

**Table 1:** Baseline characteristics of the patients

*Participants*	
Age (year)	71.4 ± 9.9
Men	27(54%)
Surgery time (min)	79.36 ± 17.57
Number of electrode implantation attempts (times)	1.87 ± 0.75
*Pacemaker indications*	
Atrioventricular block	38 (76%)
Sick sinus syndrome	4 (8%)
Heart failure with non-ischemic cardiomyopathy	8 (16%)
*Comorbid medical conditions*	
Ventricular tachycardia	4 (8%)
Hypertension	33 (66%)
Diabetes	11 (22%)
Chronic renal insufficiency	4 (8%)
Old cerebral infarction	7 (14%)
*Cardiac function indicators*	
Preoperative LVEF (%)	58.8 ± 13.9
Interventricular septal thickness (mm)	10.5 ± 1.7
Left ventricular posterior wall thickness (mm)	9.8 ± 1.3
Right ventricular posterior wall thickness (mm)	4.1 ± 0.4
Preoperative QRS interval (ms)	124.9 ± 32.6
QRS width > 150 ms	11(22%)
*Postoperative QRS interval (ms)*	113.4 ± 17.1

*LVEF* left ventricular ejection fraction, *DDD* dual chamber pacemaker, *ICD* implantable cardioverter-defibrillator

### Distribution position of the pacing electrodes with the nine-partition grid method

**Fig. 3:**
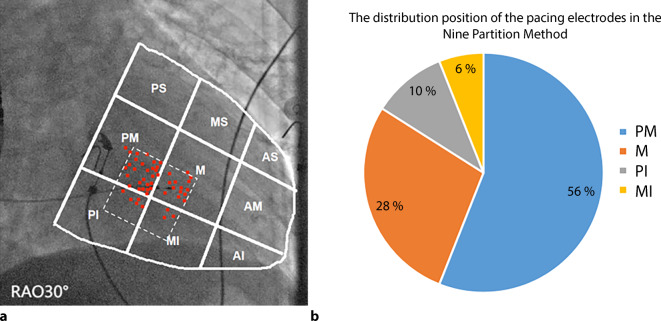
**a** RAO 30° fluoroscopic image of the whole ventricle, the *red dots* represent the distribution of the tips of LBBAP electrodes, and the *white dotted rectangle* is composed of the central points of the PM, M, PI, and MI positions. **b** Pie chart of the distribution of the electrodes in each position. *PS* posterior superior, *MS* middle superior, *AS* anterior superior, *PM* posterior median, *M* median, *AM* anterior median, *PI* posterior inferior, *MI* middle inferior, *AI* anterior inferior

As shown in Fig. 3, in the RAO 30° fluoroscopic image of the whole ventricle, the red dots represent the distribution of the tips of LBBAP electrodes in 50 patients. Among these, 28 pacing electrodes were in the PM position, 14 in the M position, 5 in the PI position, and 3 in the MI position. All pacing electrodes were located in the PM, M, PI, and MI positions or in the junction area of the above positions. Overall, 84% of the electrodes were located in the PM and M positions.

### Vertical distance from the tip of the pacing electrode to the coronary artery

Table 2 show the vertical distances from the end-systolic LBBAP electrode to the coronary artery. The farther the electrodes from the coronary arteries, the safer the coronary arteries. Damage to coronary arteries and related structures from pacemaker and implantable cardioverter-defibrillator lead implantation is a rarely reported complication that can lead to myocardial infarction and cardiac tamponade, which may occur acutely or even years later. For the LAD branch, the pacing electrodes in the M position had a greater injury risk than the pacing electrode in the MI position (15.20 ± 6.61 mm vs. 23.82 ± 9.43 mm). Like the PD branch, the pacing electrode in the PI position had a greater injury risk than the electrode in the PM position (18.96 ± 10.74 mm vs. 28.47 ± 7.67 mm). For the PL branch, the pacing electrode in the PI position had a greater injury risk than the electrode in the PM position (14.60 ± 5.26 mm vs. 23.82 ± 7.64 mm). The distance from the PM to all of the aforementioned coronary arteries was almost always at its maximum, where the electrode was safest for the coronary arteries.

**Table 2:** Distance from the electrode tip of the different positions to each coronary artery

	Mean (mm)	SD	Min (mm)	Max (mm)
*Distance from the electrode to the LAD*				
PM	21.21	9.66	5.36	43.07
M^a^	15.20	6.61	5.28	27.87
PI^b^	21.25	3.58	15.41	24.88
MI^b^	23.82	9.43	13.35	31.65
F	11.20	–	–	–
*p*	< 0.001	–	–	–
*Distance from the electrode to the PD*				
PM	28.47	7.67	12.22	44.11
M	24.76	6.87	11.95	38.12
PI^a,b^	18.96	10.74	9.51	34.37
MI^a^	22.02	1.47	20.34	23.06
F	14.64	–	–	–
*p*	< 0.001	–	–	–
*Distance from the electrode to the PL*				
PM	23.82	7.64	10.45	42.50
M^a^	19.06	7.67	9.00	32.63
PI^a,b^	14.60	5.26	8.69	23.10
MI^a^	16.21	3.93	11.78	19.28
F	20.40	–	–	–
*p*	< 0.001	–	–	–

*PM* posterior–median, *M* median, *PI* posterior–inferior, *MI* middle–inferior

^a^*p* < 0.05 when compared with PM

^b^Indicated *P* < 0.05 when compared M

### Myocardial injury

Compared with the preoperative level, there was a 20-fold postoperative troponin increase (*p* < 0.05), but it was still under the 99th percentile of the reference value upper line. Moreover, there was no significant change in the myocardial enzyme profile. These data indicated that the implantation of the LBBAP electrode could injure the coronary veins, some small blood vessels of the heart and myocardial tissue, but without obvious myocardial ischemia or infarction (Table 3).

**Table 3:** Markers of serum myocardial injury before and after LBBAP

	Preoperatively	Postoperatively	*p*
Troponin (ng/mL)	0.034	0.8	< 0.05
CK (U/L)	83.37	87.272	0.8598
CK-MB (ng/mL)	16.2	15.07	0.4456
LDH (U/L)	179.5	177.558	0.9206
MB (ng/mL)	112.34	92.04	0.5204

*CK* creatine kinase, *CK-MB* creatine kinase isoenzymes, *LDH* lactate dehydrogenase, *MB* myoglobin

Coronary angiography in all patients showed no lead compression of the LAD, PD, and PL by the LBBAP electrodes. There were no complications such as acute chest pain, acute myocardial infarction, cardiac tamponade, or lead fracture during or within 24 h of the LBBAP procedure.

## Discussion

The procedure of LBBAP is the most consistent way of physiological pacing [8, 12], because the distribution of the pacing electrode conforms to the physiological anatomical trend of the left bundle branch. However, it still lacks a specific safe field to locate the pacing electrode. Pang et al. [13] used cardiac computed tomography to evaluate the proximity of the pacemaker electrodes and ICD electrodes to the coronary arteries, but they did not measure the distance from the electrodes to the posterior descending artery of the right coronary artery and the posterior branch of the left ventricle of the right coronary artery; they also failed to give a safe pacing range for the electrodes. In addition, the presence of beam hardening and motion artifacts in cardiac computed tomography with metal pacing electrodes also affected the measurement accuracy [14]. In this study, we not only measured the specific distance between the pacing electrode and each coronary artery, but we also established a safe pacing range for electrode placement. Additionally, Becker et al. [4] reported that electrode compression of the coronary arteries is most likely to occur during the systolic period. Therefore, systolic CAG was used for analysis in this study.

The findings of this study revealed that the LAD branch of the coronary artery is the most susceptible to injury by the LBBAP electrode due to its shorter average distance. Specifically, using the nine-partition grid method, the electrode positioned at the M location posed the highest risk of injury to the LAD. This observation aligns with the average distance reported by Pang et al. [15], who utilized cardiac computed tomography in patients with RVP (19.69 vs. 18.9 mm). Furthermore, the study identified that the average distance from the PI position lead to the PD and PL branches was the shortest, whereas the average distance from the PM position lead to the PD and PL branches was the longest. These results suggest that the PM position is a safe area for locating the LBBAP electrode in relation to the PD and PL branches. In summary, the distance between the PM electrode and each coronary artery consistently demonstrated a relatively greater length compared with other positions, indicating that the PM position provides a safe location for pacing electrode placement.

Furthermore, the anatomical location of LBBAP electrodes makes them prone to causing injury to the ventricular septal branches. Previous research by Qi et al. [2] revealed that ventricular septal vessels were visualized during the injection of a contrast agent into the C315 His sheath when assessing the depth of electrode implantation in the LBBAP procedure. Although these vessels were identified as coronary venous vessels, their presence indicates the potential risk of injury to the ventricular septal branches during electrode placement. Therefore, in this study, endo-angiography through C315 His was used to observe the injury of the ventricular septal branches. Although 38 patients underwent endo-angiography, no development of the ventricular septal artery or hematoma formation was observed. Due to variations in diameter, length, number, and distribution of the anterior or posterior ventricular septal branches among individuals, it becomes challenging to accurately measure the precise distance between the electrode tip and a specific ventricular septal branch. Consequently, quantifying the injury risk to these branches becomes difficult. However, in cases where the LBBAP electrode is implanted close to the ventricular free wall, there is an increased risk of injury to the interventricular sulcus section of the anterior descending branch, the posterior branch of the left ventricle, and the posterior descending section of the right coronary artery. To address this potential risk, it is advisable to perform endo-angiography with CAG during the LBBAP procedure.

In addition to the risks associated with ventricular septal branches and the proximity of the electrode to the ventricular free wall, anatomical abnormalities of the coronary arteries can also contribute to vessel injury during LBBAP procedures [16]. It is important to differentiate between the compression of the LAD artery by myocardial fibers during systole, which returns to normal blood flow during diastole, and the compression of the LAD by the pacing electrode. Furthermore, it is worth noting that there were no evident symptoms such as chest pain, dynamic ST‑T changes in the ECG, or increased markers of myocardial injury during or after the LBBAP procedure. This suggests that the procedure did not result in significant myocardial damage or ischemia.

### Limitations

However, there are several limitations to consider in our study. Firstly, the detection of vein injuries accompanying the coronary arteries using CAG may be challenging [17]. We used endo-angiography through C315 His to visualize the ventricular septal artery; however, due to the variations in the number, thickness, and distribution of ventricular septal arteries among individuals, it was challenging to measure the precise distance between the electrode and a standard ventricular septal vessel. Therefore, selecting a standardized vessel for measurement purposes proved difficult. Secondly, the assessment of small blood vessel injuries and myocardial injury presented challenges in our study. Traditional methods such as serum troponin levels, myocardial enzyme profiles, and ECG may not be sensitive enough to detect subtle injuries [14]. Although our study observed a more than 20-fold increase in serum troponin levels compared to preoperative levels, it did not reach the threshold for diagnosing myocardial infarction. It is worth noting that factors other than myocardial infarction, such as endurance exercise, can also lead to an increase in cardiac troponin levels [18]. Thirdly, our study focused on pacing electrodes positioned in the PM, M, MI, and PI locations. Therefore, the safety and potential risks associated with pacing electrodes in other positions were not assessed. Lastly, the small size of our study population may introduce sample bias and limit the generalizability of the findings. Further studies involving larger and more diverse populations are necessary to validate and expand upon our results.

## Conclusion

This study provides compelling evidence to support the notion that use of the left bundle branch area pacing (LBBAP) electrode carries a higher inherent risk of causing injury to the left anterior descending branch artery (LAD) when compared with the posterior descending branches (PD) and posterior ventricular branches (PL). However, LBBAP electrodes located in the rectangle formed by connecting the central points of the posterior–middle, median, posterior–inferior, and middle–inferior in right anterior oblique 30° projection are relatively safe for the coronary arteries. Especially, the electrode in the PM position has a lower risk of injury to the coronary arteries.

## Data Availability

All data generated or analyzed during this study are included in this article. Further enquiries can be directed to the corresponding author. Left bundle branch area pacing (LBBAP) involves the insertion of electrodes that traverse from the right ventricular septum to the left ventricular septum subendocardium. This technique carries a potential risk of damaging the coronary arteries and veins, including the ventricular septal branches, the left anterior descending branch (LAD), the right posterior coronal descending branch (PD), and the posterior left ventricular branch (PL). To assess this risk, we employed the nine-partition grid method to measure the distance between the electrode tip and the coronary arteries using fluoroscopic images. Our study yielded noteworthy results, indicating that LBBAP electrodes positioned in the posterior median (PM) location exhibited a reduced likelihood of injuring the coronary arteries. This finding holds significant clinical implications as it suggests that careful placement of LBBAP electrodes in the PM position can minimize the risk of complications related to coronary artery injury. LBBAP has emerged as a promising physiological pacing technique that offers several advantages over traditional pacing methods such as right ventricular pacing (RVP) and His bundle pacing (HBP). LBBAP has been found to effectively reduce the width of the QRS complex, synchronize the mechanical contraction of the left ventricular myocardium, improve cardiac function, and provide resistance against atrial fibrillation and heart failure [1]. One of the key features of LBBAP is the placement of electrodes, which requires permeation from the right ventricular septum to the left ventricular septum subendocardium. Therefore, there is a potential risk of injuring the coronary arteries and veins, such as the ventricular septal branches, the LAD, the right PD, and PL. Furthermore, Qi et al. [2] found that the pacemaker electrode penetrated the interventricular septal vessels, which were successfully visualized upon injecting the contrast medium into the C315 His sheath. And some studies have found that ventricular septal pacing electrodes can compress the anterior descending artery to different degrees, resulting in varying degrees of myocardial infarction [3–7]. However, to our knowledge, there are no studies concerning LBBAP electrodes and coronary vascular injury. Therefore, this study aimed to evaluate the risk of intraoperative coronary injury by measuring the distance between the tip of LBBAP electrodes and the coronary arteries to provide a reliable clinical reference for the safety of the LBBAP procedure.

## Declarations

For this article no studies with human participants or animals were performed by any of the authors. All studies mentioned were in accordance with the ethical standards indicated in each case.
